# Development and Validation of a Lateral Flow Immunoassay for Rapid Detection of NDM-Producing Enterobacteriaceae

**DOI:** 10.1128/JCM.00248-17

**Published:** 2017-06-23

**Authors:** Hervé Boutal, Thierry Naas, Karine Devilliers, Saoussen Oueslati, Laurent Dortet, Sandrine Bernabeu, Stéphanie Simon, Hervé Volland

**Affiliations:** a Service de Pharmacologie et Immunoanalyse, CEA, INRA, Université Paris-Saclay, Gif-sur-Yvette, France; b EA7361, Université Paris-Sud, Université Paris-Saclay, LabEx Lermit, Bacteriology-Hygiene Unit, APHP, Hôpital Bicêtre, Le Kremlin-Bicêtre, France; c Associated French National Reference Center for Antibiotic Resistance: Carbapenemase-Producing Enterobacteriaceae, Le Kremlin-Bicêtre, France; Memorial Sloan Kettering Cancer Center

**Keywords:** NDM-1, lateral flow immunoassay, detection

## Abstract

The global spread of carbapenemase-producing Enterobacteriaceae (CPE) that are often resistant to most, if not all, classes of antibiotics is a major public health concern. The NDM-1 carbapenemase is among the most worrisome carbapenemases given its rapid worldwide spread. We have developed and evaluated a lateral flow immunoassay (LFIA) (called the NDM LFIA) for the rapid and reliable detection of NDM-like carbapenemase-producing Enterobacteriaceae from culture colonies. We evaluated the NDM LFIA using 175 reference enterobacterial isolates with characterized β-lactamase gene content and 74 nonduplicate consecutive carbapenem-resistant clinical isolates referred for expertise to the French National Reference Center (NRC) for Antibiotic Resistance during a 1-week period (in June 2016). The reference collection included 55 non-carbapenemase producers and 120 carbapenemase producers, including 27 NDM producers. All 27 NDM-like carbapenemase producers of the reference collection were correctly detected in less than 15 min by the NDM LFIA, including 22 strains producing NDM-1, 2 producing NDM-4, 1 producing NDM-5, 1 producing NDM-7, and 1 producing NDM-9. All non-NDM-1 producers gave a negative result with the NDM LFIA. No cross-reaction was observed with carbapenemases (VIM, IMP, NDM, KPC, and OXA-48-like), extended-spectrum β-lactamases (ESBLs) (TEM, SHV, and CTX-M), AmpCs (CMY-2, DHA-2, and ACC-1), and oxacillinases (OXA-1, -2, -9, and -10). Similarly, among the 74 referred nonduplicate consecutive clinical isolates, all 7 NDM-like producers were identified. Overall, the sensitivity and specificity of the assay were 100% for NDM-like carbapenemase detection with strains cultured on agar. The NDM LFIA was efficient, rapid, and easy to implement in the routine workflow of a clinical microbiology laboratory for the confirmation of NDM-like carbapenemase-producing Enterobacteriaceae.

## INTRODUCTION

Enterobacteriaceae have a major role as causes of nosocomial infections (and, for Escherichia coli, also of community-acquired infections), and expanded-spectrum cephalosporins and carbapenems are essential in the treatment of these infections (1). The dissemination of broad-spectrum β-lactamases (extended-spectrum β-lactamases [ESBLs] and carbapenemases) among Enterobacteriaceae is undoubtedly a matter of great public health concern. Indeed, ESBL-producing Enterobacteriaceae are resistant to all β-lactams up to third-generation cephalosporins (2), and this leads to the use of last-resort antibiotics such as carbapenems. Unfortunately, the emergence of carbapenemase-producing Enterobacteriaceae (CPE), which are often resistant to several antibiotic classes, has become a major issue, as they are often involved in nosocomial or community-acquired infections (3).

Of the carbapenemases most commonly encountered (4), the metallo-β-lactamase (MBL) NDM-1 (Ambler class B) (5) shows the most tremendous spread (3, 6). Moreover, the *bla*_NDM-1_ gene is located on plasmids that encode other resistance determinants, conferring a broad drug resistance pattern (7). Initially isolated in India, it is now endemic in the entire Indian subcontinent and represents a major threat considering the high rate of acquisition (72.4%) of multiresistant Enterobacteriaceae (MRE), including NDM-1-expressing strains, while traveling in this area (8). Thus, the rapid and reliable detection of NDM-1-producing bacteria is essential to identify infected or colonized patients in order to prevent further spread and to provide proper treatments.

Tests to detect carbapenemases have already been developed. Some are based on the detection of carbapenem hydrolysis using matrix-assisted laser desorption ionization–time of flight mass spectrometry (MALDI-TOF MS) (9, 10). Others are biochemical tests (e.g., Carba NP test and derivatives [11, 12] and the carbapenem inactivation method [CIM] [13]) or phenotypic tests like the OXA-48 disk test (14, 15) and various phenotypic confirmation tests, including tests of inhibition of carbapenemase activity (15, 16). Some of these tests have proven useful albeit with turnaround times of 2 to 24 h (14, 15). These tests may be used on colonies, and some have been evaluated directly on blood cultures (17). Recently, a lateral flow immunoassay (LFIA) for the detection of OXA-48-type carbapenemases has been evaluated (18). This test yields results from cultured strains within 15 min, with 100% specificity and sensitivity. Finally, molecular methods, such as endpoint PCR and real-time PCR, can also be used in single or multiplex formats targeting the main carbapenemases, with high specificity and sensitivity (19–21). However, these methods are expensive and require a high level of expertise to obtain accurate results.

To respond to the needs optimally, antimicrobial drug resistance detection methods must be cheap (reduced costs of consumables and equipment) and easy to use (reduced technical complexity) for the end user. This led us to develop an LFIA for the detection of NDM-like carbapenemases that presents several advantages: robust technology, easily transferable in a commercialized version, stable for more than 24 months without refrigeration, user-friendly (no requirement for trained staff), high performance (sensitive and specific), low cost (around 6€ per test [22, 23], compared to more than 20€ for molecular tests), and detection results in less than 30 min without the need for highly technical equipment for the readout. We have validated this assay on 175 agar-cultured enterobacterial isolates (27 expressing NDM-like enzymes), with test results in less than 15 min, 100% sensitivity, and 100% specificity.

## RESULTS

### Combinatorial test and best-pair selection.

**FIG 1:**
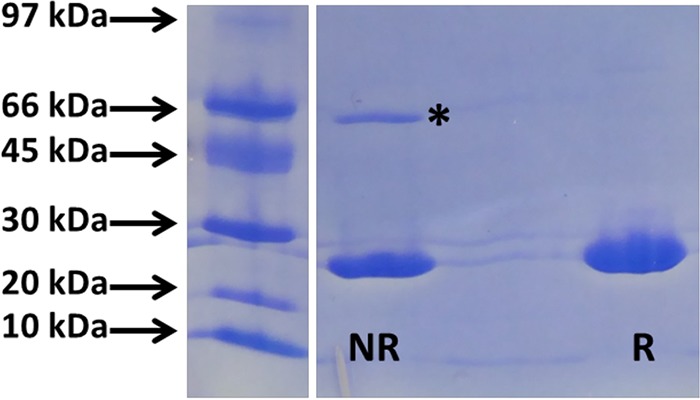
SDS-PAGE (Pharmacia Phast system) with purified recombinant NDM-1 (2 mg/ml) and Coomassie blue staining. NR, nonreducing conditions; R, reducing conditions. * indicates dimers.

**FIG 2:**
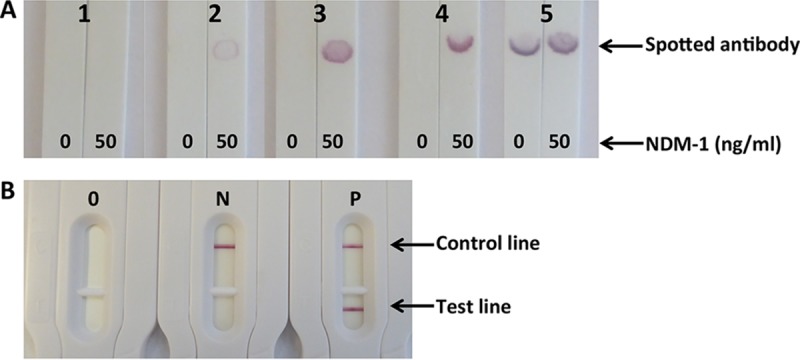
(A) Pair selection with handmade strips. Shown are signals obtained with 0 and 50 ng/ml of recombinant NDM-1 as a function of the pair performance. 1, no specific signal; 2, low specific signal; 3, medium specific signal; 4, high specific signal (U shape); 5, high specific and nonspecific signals. (B) Manufactured cassettes. O, cassette before use; N, negative result; P, positive result.

Eighty milligrams of recombinant NDM was produced from 1 liter of culture. SDS-PAGE (Fig. 1) under nonreducing conditions showed a major band corresponding to the theoretical molecular mass of 26 kDa and another one close to 66 kDa. NDM-1 can exist as a monomer and dimer in solution (24), and only a band corresponding to monomers is observed under reducing conditions. The presence of dimers was also confirmed by trypsin digestion and MALDI-TOF analysis. This protein was then used to immunize mice. Twenty-two monoclonal antibodies (MAbs) were finally selected (named NDM 101 to NDM 122). A total of 484 pairs were tested during the combinatorial study done with spotted strips. The 3 pairs of antibodies displaying the strongest specific signal with a specific U-shaped signal (Fig. 2A) revealing a high-affinity capture antibody (25) and no nonspecific signals were further selected (NDM 105/NDM 103, NDM 105/NDM 106, and NDM 105/NDM 120). In order to decide between these three pairs, they were further tested with serial dilutions of NDM-expressing strains. The NDM 105/NDM 103 pair, showing the lowest limit of detection, was selected, and a batch of 1,000 tests (strip plus cassette) was produced (NG Biotech) in order to carry out assay validation (sensitivity and specificity) (Fig. 2B).

### Limit of detection using the NDM LFIA.

**FIG 3:**
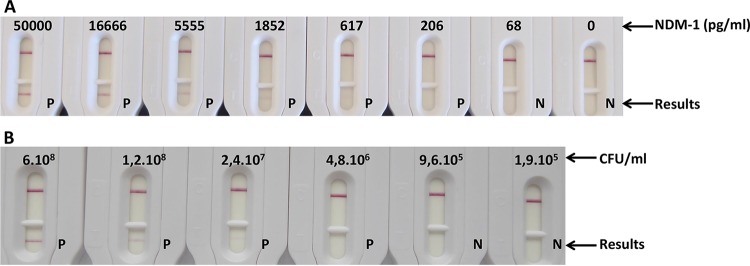
Limit of detection in extraction buffer. (A) Serial dilutions with recombinant NDM-1 (nanograms per milliliter). (B) Serial dilution with NDM-1-expressing Klebsiella pneumoniae (CFU per milliliter). P, positive result; N, negative result.

Although a positive line test was visible (by the naked eye) at 208 pg/ml (equivalent to 5 × 10^6^ CFU/ml), it does not appear on the picture due to the camera sensitivity. Thus, limits of detection (LOD) of the NDM LFIA of between 208 and 617 pg/ml and around 5 × 10^6^ CFU/ml were determined by eye after 15 min of migration using purified recombinant NDM protein or one NDM-1-producing Klebsiella pneumoniae strain (Fig. 3).

### Performance of the NDM LFIA with reference isolates.

**FIG 4:**
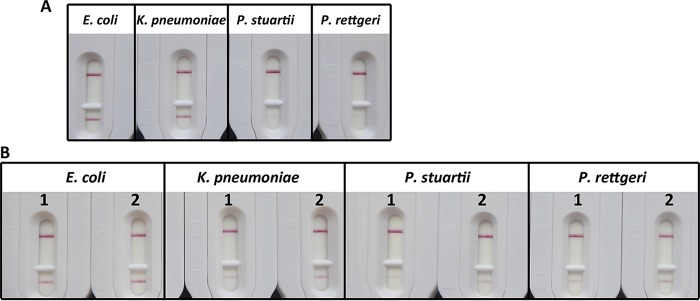
(A) Results obtained with different strains (colonies of equal sizes were tested). 1, NDM-4-expressing Escherichia coli; 2, NDM-1-expressing Klebsiella pneumoniae; 3, NDM-1-expressing Providencia stuartii; 4, NDM-1-expressing Providencia rettgeri. (B) Extraction buffer performance. For Escherichia coli and Klebsiella pneumoniae, 1 was colony suspended in 1 ml. For Providencia stuartii and Providencia rettgeri, 10 colonies were suspended in 300 μl. The suspension was separated in two parts. 1, extraction buffer only; 2, extraction buffer plus 5 freeze-thaw cycles and 1 min of sonication (14 W).

As shown in Table 1, the NDM LFIA was able to detect all 27 reference strains expressing NDM-like enzymes (22 expressing NDM-1, 2 expressing NDM-4, 1 expressing NDM-5, 1 expressing NDM-7, and 1 expressing NDM-9). All the strains that did not produce an NDM carbapenemase gave negative results. This corresponded to 30 strains expressing class A carbapenemases (22 expressing KPC, 3 expressing IMI, 1 expressing NMC-A, 1 expressing SME, 1 expressing GES, and 1 expressing FRI-1), 28 strains expressing class B carbapenemases (17 expressing VIM, 11 expressing IMP, and 1 expressing GIM), 40 strains expressing class D carbapenemases (21 expressing OXA-48, 1 expressing OXA-162, 9 expressing OXA-181, 5 expressing OXA-204, 2 expressing OXA-232, and 2 expressing OXA-244), and 55 non-carbapenemase producers (Table 1). Our results perfectly correlated with the β-lactamase genotype of the strains as determined by PCR analysis. Positive results showed a dark pink band (Fig. 4A) in most cases, except for Providencia rettgeri and Providencia stuartii. These two strains were detected, but the corresponding tests showed signals with a much lower intensity (Fig. 4A). This validation experiment showed that our test was able to detect NDM-producing bacteria with 100% sensitivity and 100% specificity (i.e., without any false-negative or false-positive results).

**TABLE 1:** Results of the NDM LFIA and the Carba NP test in a collection of strains comprising carbapenemase and non-carbapenemase producers

β-Lactam resistance mechanism	Bacterial species	No. of isolates	β-Lactamase content (PCR)^*b*^	Result^*a*^	
				NDM LFIA	Carba NP test
Ambler class B carbapenemases					
NDM type	E. coli	1	NDM-1 + OXA-1 + OXA-10 + CMY-16 + TEM-1	P	P
	E. coli	1	NDM-1 + OXA-1 + TEM-1	P	P
	E. coli	1	NDM-1 + CTX-M-15 + TEM-1	P	P
	E. coli	1	NDM-1 + OXA-1 + OXA-2 + CTX-M-15 + TEM-1	P	P
	E. coli	1	NDM-1 + CTX-M-15 + TEM-1	P	P
	E. coli	1	NDM-4 + CTX-M-15 + OXA-1	P	P
	E. coli	1	NDM-4 + CTX-M-15 + CMY-6	P	P
	E. coli	1	NDM-5 + TEM-1 + CTX-M-15	P	P
	E. coli	1	NDM-6 + CTX-M-15 + OXA-1	P	P
	E. coli	1	NDM-7 + CTX-M-15	P	P
	K. pneumoniae	1	NDM-1 + CTX-M-15 + SHV-11 + OXA-1	P	P
	K. pneumoniae	1	NDM-1 + CTX-M-15 + CMY-4 + OXA-1	P	P
	K. pneumoniae	1	NDM-1 + CTX-M-15 + OXA-1 + OXA-9 + TEM-1 + SHV-28 + SHV-11	P	P
	K. pneumoniae	1	NDM-1 + OXA-1 + SHV-11	P	P
	K. pneumoniae	1	NDM-1 + OXA-1 + CTX-M-15 + TEM-1 + SHV-28 + OXA-9 + CMY-6	P	P
	K. pneumoniae	1	NDM-1 + TEM-1 + CTX-M-15 + SHV-12 + OXA-9	P	P
	K. pneumoniae	1	NDM-1 + TEM-1 + CTX-M-15 + SHV-12 + OXA-9	P	P
	K. pneumoniae	1	NDM-1 + TEM-1 + CTX-M-15 + SHV-11 + OXA-1	P	P
	P. stuartii	1	NDM-1 + OXA-1 + CMY-6 + TEM-1	P	P
	P. rettgeri	1	NDM-1 + CTXM-15	P	P
	Salmonella enterica	1	NDM-1 + CTX-M-15 + TEM-1 + OXA-1 + OXA-9 + OXA-10	P	P
VIM type	E. coli	1	VIM-1	N	P
	E. coli	1	VIM-1 + CMY-13	N	P
	E. coli	1	VIM-4	N	P
	K. pneumoniae	3	VIM-1	N	P
	K. pneumoniae	4	VIM-1 + SHV-5	N	P
	K. pneumoniae	1	VIM-1 + SHV-12	N	P
	K. pneumoniae	1	VIM-1 + TEM-1 + SHV-5	N	P
	K. pneumoniae	1	VIM-19 + CTX-M-3 + TEM-1 + SHV-1	N	P
	Enterobacter cloacae	1	VIM-1 + SHV-70	N	P
	E. cloacae	1	VIM-4 + TEM-1 + SHV-31	N	P
	Citrobacter freundii	1	VIM-2 + TEM-1 +	N	P
	C. freundii	1	VIM-2 + TEM-1 + OXA-9 + OXA-10	N	P
IMP type	E. coli	1	IMP-1	N	P
	E. coli	1	IMP-8 + SHV-12	N	P
	K. pneumoniae	1	IMP-1	N	P
	K. pneumoniae	1	IMP-1 + TEM-15	N	P
	K. pneumoniae	1	IMP-1 + TEM-15	N	P
	K. pneumoniae	1	IMP-1 + SHV-5	N	P
	K. pneumoniae	1	IMP-8	N	P
	K. pneumoniae	1	IMP-8 + SHV-12	N	P
	E. cloacae	1	IMP-8	N	P
	E. cloacae	1	IMP-8 + SHV-12	N	P
	Serratia marcescens	1	IMP-11	N	P
GIM type	E. cloacae	1	GIM-1	N	P
Ambler class A carbapenemases					
KPC-2	E. coli	1	KPC-2	N	P
	E. coli	1	KPC-2 + CTXM-15	N	P
	E. coli	1	KPC-2 + TEM-1 + OXA-9	N	P
	E. coli	1	KPC-2 + CTX-M-9 + TEM-1	N	P
	K. pneumoniae	1	KPC-2 + SHV-11 + TEM-1 + CTX-M-2	N	P
	K. pneumoniae	1	KPC-2 + SHV-11 + TEM-1 + CTX-M-2 + OXA-9	N	P
	K. pneumoniae	1	KPC-2 + SHV-11	N	P
	K. pneumoniae	1	KPC-2 + TEM-1 + SHV-1 + CTX-M-15	N	P
	K. pneumoniae	1	KPC-2 + SHV-11 + TEM-1 + SHV-12 + OXA-9	N	P
	K. pneumoniae	1	KPC-2 + SHV-11	N	P
	K. pneumoniae	1	KPC-2 + SHV-11 + TEM-1	N	P
	E. cloacae	1	KPC-2	N	P
	E. cloacae	1	KPC-2 + TEM-1	N	P
	E. cloacae	1	KPC-2 + TEM-1 + OXA-1 + CTX-M-15	N	P
	E. cloacae	1	KPC-2 + TEM-1 + SHV-11	N	P
	E. cloacae	1	KPC-2 + TEM-3	N	P
	C. freundii	1	KPC-2 + TEM-1	N	P
	S. marcescens	1	KPC-2 + TEM-1 + SHV-12	N	P
	S. marcescens	1	KPC-2 + TEM-1	N	P
KPC-3	K. pneumoniae	1	KPC-3 + TEM-1 + SHV-1 + OXA-9	N	P
	K. pneumoniae	1	KPC-3 + SHV-11 + OXA-9 + TEM-1	N	P
	Klebsiella ozaenae	1	KPC-3 + OXA-9 + TEM-1	N	P
IMI type	E. cloacae	1	IMI-1	N	P
	Enterobacter asburiae	1	IMI-2	N	P
	E. asburiae	1	IMI-2	N	N
NmcA	E. cloacae	1	NmcA	N	P
Sme type	S. marcescens	1	Sme-1	N	P
	S. marcescens	1	Sme-2	N	P
GES type	E. cloacae	1	GES-5	N	N
FRI	E. cloacae	1	FRI-1	N	P
Ambler class D carbapenemases					
OXA-48	E. coli	4	OXA-48 + CTX-M-15	N	P
	E. coli	1	OXA-48 + CTX-M-24 + TEM-1	N	P
	E. coli	1	OXA-48 + TEM-1 + CTX-M-1	N	P
	K. pneumoniae	4	OXA-48	N	P
	K. pneumoniae	1	OXA-48 + CTX-M-15	N	P
	K. pneumoniae	1	OXA-48 + TEM-1	N	P
	K. pneumoniae	1	OXA-48 + SHV-11	N	P
	K. pneumoniae	2	OXA-48 + CTX-M-15 + TEM-1	N	P
	E. cloacae	2	OXA-48 + TEM-1 + CTX-M-15 + OXA-1	N	P
	E. cloacae	1	OXA-48 + SHV-5	N	P
	Citrobacter koseri	1	OXA-48	N	P
	C. koseri	1	OXA-48 + TEM-1	N	P
	C. freundii	1	OXA-48 + SHV-12 + TEM-1	N	P
OXA-162	K. pneumoniae	1	OXA-162 + TEM-1 + SHV-11 + CTX-M-15	N	P
OXA-181	E. coli	1	OXA-181	N	P
	E. coli	1	OXA-181	N	P
	K. pneumoniae	1	OXA-181 + SHV-11 + TEM-1 + CTX-M-15 + NDM-1 + OXA-1	P	P
	K. pneumoniae	1	OXA-181 + SHV-27 + CTX-M-15 + TEM-1 + NDM-1 + OXA-1	P	P
	K. pneumoniae	1	OXA-181 + SHV-11 + CTX-M-15 + NDM-1 + OXA-1	P	P
	K. pneumoniae	1	OXA-181 + SHV-11 + TEM-1 + CTX-M-15 + NDM-1 + OXA-9	P	P
	K. pneumoniae	1	OXA-181 + SHV-11 + CTX-M-15 + OXA-1	N	P
	K. pneumoniae	1	OXA-181 + NDM-1 + SHV-2 + CTX-M-15 + OXA-1	P	P
	C. freundii	1	OXA-181 + NDM-1 + OXA-1 + OXA-9 + OXA-10 + CTX-M-15 + TEM-1	P	P
OXA-204	K. pneumoniae	1	OXA-204 + CMY-4	N	P
	E. coli	1	OXA-204 + CMY-2 + CTX-M-15 + OXA-1	N	P
	E. coli	1	OXA-204 + CMY-4+ CTX-M-15 + OXA-1	N	P
	E. coli	1	OXA-204 + CMY-4 + CTX-M-15	N	P
	K. pneumoniae	1	OXA-204 + SHV-28 + TEM-1 + CTX-M-15	N	P
OXA-232	E. coli	1	OXA-232 + CTX-M-15 + OXA-1	N	P
	K. pneumoniae	1	OXA-232 +SHV-1 + TEM-1 + CTX-M-15 + OXA-1	N	P
OXA-244	E. coli	1	OXA-244 + TEM-1 + CMY-2	N	E
	E. coli	1	OXA-244 + TEM-1 + CMY-2	N	N
Non-carbapenemase producers					
Wild type	K. pneumoniae	1	SHV-11	N	N
Acquired cephalosporinase	E. coli	1	DHA-1	N	N
	E. coli	1	ACC-1	N	N
	K. pneumoniae	1	DHA-2	N	N
	Proteus mirabilis	1	ACC-1	N	N
ESBL	E. coli	1	CTX-M-1	N	N
	E. coli	1	CTX-M-3	N	N
	K. pneumoniae	1	CTX-M-3	N	N
	E. coli	2	CTX-M-14	N	N
	K. pneumoniae	1	CTX-M-14	N	N
	E. coli	2	CTX-M-15	N	N
	K. pneumoniae	3	CTX-M-15	N	N
	E. cloacae	1	CTX-M-15	N	N
	E. cloacae	1	VEB-1	N	N
Cephalosporinase + impermeability	E. coli	1	➚➚➚ cephalosporinase	N	N
	E. cloacae	13	➚➚➚ cephalosporinase	N	N
	E. cloacae	1	➚➚➚ cephalosporinase + CTX-M-15	N	N
	Enterobacter aerogenes	1	➚➚➚ cephalosporinase	N	N
	Morganella morganii	1	➚➚➚ cephalosporinase	N	N
ESBL + impermeability	E. coli	1	CTX-M-15	N	N
	K. pneumoniae	1	CTX-M-15 + SHV-1	N	N
	K. pneumoniae	2	CTX-M-15 + TEM-1 + SHV-1	N	N
	K. pneumoniae	1	CTX-M-15 + SHV-11	N	N
	K. pneumoniae	1	CTX-M-15 + SHV-28 - TEM-1	N	N
	K. pneumoniae	1	TEM-1 + SHV-28	N	N
	K. pneumoniae	4	CTX-M-15 + TEM-1 + SHV-11	N	N
	K. pneumoniae	1	CTX-M-15 + TEM-1 + SHV-12	N	N
	K. pneumoniae	1	CTX-M-15 + TEM-1 + SHV-1 + OXA-1	N	N
ESBL + cephalosporinase + impermeability	E. cloacae	3	➚➚➚ case + CTX-M-15	N	N
	C. freundii	1	➚➚➚ case +TEM-3	N	N
Extended-spectrum oxacillinases	K. pneumoniae	1	OXA-163	N	N
	E. cloacae	1	OXA-163	N	N
	S. marcescens	1	OXA-405	N	N

a P indicates a positive result, N indicates a negative result, and E indicates an equivocal result.

b ➚➚➚ indicates a hyperproduced cephalosporinase.

### Effects of growth media on LFIA results.

Ten strains (6 NDM producers and 4 non-NDM producers) from the reference collection were grown on 7 of the most common media used for bacteria growth (Table 2). Some media currently used for the identification and/or selection of carbapenemase-expressing strains generate colonies with genus-specific colors (blue, green, pink, or dark purple on Uri-4 plates, for example). These colored colonies, suspended in extraction buffer, stained the latter in a similar manner. This staining did not interfere with the test results, since the 6 NDM producers gave positive results and the 4 non-NDM producers gave negative results, whatever the medium used for culture. Thus, colony staining did not modify the appearance of the nitrocellulose membrane and still yielded easily interpretable results (data not shown).

**TABLE 2:** Results of the NDM LFIA with strains grown on different agar media

Bacterial species	No. of isolates	β-Lactamase content (PCR)	Result with culture medium^*a*^						
			MH	TSA	COH	Uri-4	DRIG	ChromID ESBL	Carba Smart
E. coli	1	NDM-1 + OXA-1 + OXA-2 + CTX-M-15 + TEM-1	P	P	P	P	P	P	P
E. coli	1	NDM-1 + CTX-M-15 + TEM-1	P	P	P	P	P	P	P
K. pneumoniae	1	NDM-1 + CTX-M-15 + SHV-11 + OXA-1	P	P	P	P	P	P	P
K. pneumoniae	1	NDM-1 + CTX-M-15 + CMY-4 + OXA-1	P	P	P	P	P	P	P
K. pneumoniae	1	NDM-1 + CTX-M-15 + OXA-1 + OXA-9 + TEM-1 + SHV-28 + SHV-11	P	P	P	P	P	P	P
Salmonella enterica	1	NDM-1 + CTX-M-15 + TEM-1 + OXA-1 + OXA-9 + OXA-10	P	P	P	P	P	P	P
E. coli	1	VIM-1 + CMY-13	N	N	N	N	N	N	N
K. pneumoniae	1	VIM-1	N	N	N	N	N	N	N
E. cloacae	1	VIM-4 + TEM-1 + SHV-31	N	N	N	N	N	N	N
S. marcescens	1	IMP-11	N	N	N	N	N	N	N

a MH, Mueller-Hinton agar; TSA, Trypticase soya agar; COH, Columbia agar plus 5% horse blood; DRIG, Drigalski agar; Uri-4, URISelect 4 medium; Carba Smart, ChromID Carba Smart; P, positive result; N, negative result.

### Prospective evaluation of the NDM LFIA.

Using PCR and Carba NP tests, a carbapenemase was detected in 44% (33/74) of the isolates. Subsequent sequencing of the positive PCR products allowed the identification of the variants of a given carbapenemase (Table 3). Concerning the 41 non-carbapenemase-producing isolates, the decreased carbapenem susceptibility might result from ESBL and/or cephalosporinase production associated with reduced outer membrane permeability (not evaluated).

**TABLE 3:** Results of a prospective study of the NDM LFIA in comparison with PCR and the Carba NP test in enterobacterial isolates with reduced susceptibility referred to the French NRC

β-Lactam resistance mechanism	Bacterial species	No. of isolates	β-Lactamase content (PCR)	Result^*a*^	
				NDM LFIA	CarbaNP test
Carbapenemases producers					
NDM type	E. coli	1	NDM-like	P	P
	K. pneumoniae	1	NDM-like	P	P
	E. cloacae	1	NDM-like	P	P
OXA-48-like	E. coli	8	OXA-48-like	N	P
	E. coli	1	NDM-like + OXA-48-like	P	P
	K. pneumoniae	5	OXA-48-like	N	P
	E. cloacae	1	OXA-48-like	N	P
KPC	K. pneumoniae	2	KPC-like	N	P
VIM	E. cloacae	1	VIM-like	N	P
Non-carbapenemase producers	E. coli	2		N	N
	K. pneumoniae	8		N	N
	E. cloacae	7		N	N
	Klebsiella oxytoca	1		N	N
	Hafnia alvei	1		N	N
	S. marcescens	1		N	N
	C. freundii	1		N	N

a P indicates a positive result, and N indicates a negative result.

The test was able to detect all isolates producing an NDM carbapenemase (7 isolates corresponding to 2 NDM-1 and 5 NDM-5 producers). All isolates that did not produce an NDM-like carbapenemase gave negative results. These isolates included 26 carbapenemase producers (23 expressing OXA-48-like enzymes, 2 expressing KPC-like enzymes, and 1 expressing a VIM-like enzyme) and the 41 non-carbapenemase-producing Enterobacteriaceae (Table 3).

## DISCUSSION

The spread of NDM-expressing strains is a major public health concern. From an infection control standpoint, the rapid identification of such strains is essential. A fast and easy-to-use test like the NDM LFIA described here would be a valuable tool to identify such producers and stratify the carriers.

During the development of the NDM LFIA, all the screening steps until the selection of 22 MAbs were done by using an immunoenzymatic assay to test the capacity of MAbs to bind recombinant NDM-1. The assay conditions of this screening format (see Text S1 in the supplemental material) are very different from those of LFIA (duration of the assay, contact of antibodies with antigen, buffers, washing steps, etc.) and are not predictive of antibody performances in LFIAs (25). In order to select the MAbs best adapted to the LFIA, we performed a combinatorial analysis by spotting the MAb onto the membrane so as to be closer to the operating conditions used in the LFIA format. As we had previously observed that the best pair for the detection of a recombinant protein is not systematically the best for the detection of the natural protein, the final selection was performed by using serial dilutions of strains expressing NDM-like enzymes. This resulted in the selection of one pair of MAbs, NDM 105 as the capture antibody and NDM 103 as the colloidal gold reporter antibody, for the validation experiments. The LOD of this LFIA is close to those of other similar tests developed in our laboratory for Staphylococcus enterotoxin B (SEB) (312 pg/ml) and Yersinia spp. (range from 10^5^ CFU/ml to 10^6^ CFU/ml), for example (26, 27). Moreover, a commercial LFIA for OXA-48 detection (23) has a comparable LOD of 2.41 × 10^6^ CFU/ml.

The validation performed with well-characterized clinical strains showed that the NDM LFIA is able to detect NDM-1 and all its variants available at the French National Reference Center (NRC), whether or not associated with other β-lactamases. No false-positive, no false-negative, and no ambiguous results were observed with the 249 tested isolates. Our test therefore showed 100% sensitivity and 100% specificity. When grown on chromogenic plates currently used in laboratories to detect CRE (such as ChromID Carba; bioMérieux, Marcy l'Etoile, France) or simply enterobacterial species in urine samples (such as ChromID CPS [bioMérieux] and Uri-4 [Bio-Rad, Marnes la Coquette, France]), bacterial isolates may show a very strong coloration (for example, dark blue for Klebsiella). Nevertheless, this coloration did not lead to false-positive results in the NDM LFIA. Thus, our assay is fully compatible with most samples handled in bacteriology laboratories. Our assay is easy to use and does not require any specific equipment or skills. The results are easy to read after 15 min of migration. However, for most positive samples, the positivity of NDM carbapenemase-producing strains could already be evidenced after 1 or 2 min. This test could be relevant in areas with a high prevalence of NDM producers in enterobacterial isolates with decreased susceptibility to carbapenems and in strains grown on CPE-screening media. Providencia stuartii and P. rettgeri showed positive results with a lower intensity. This could be due to the location of the *bla*_NDM-1_ gene. A chromosomal or plasmid location under the control of a promoter with low efficiency in these strains could result in a low expression level. Partial extraction was observed for Providencia strains and also for Klebsiella (Fig. 4B), but this inconvenience seems minor, as NDM-1 expression in Klebsiella was strongly detected. The fact that more bacteria were needed to obtain an equivalent signal, as with our NDM-expressing E. coli or K. pneumoniae strains (Fig. 4B), could confirm the major influence of the expression level.

Besides these good results, we have to keep in mind that any new mutation occurring in the epitope of one of the MAbs involved may give false-negative results. To face this eventuality, we have already identified 4 compatibility groups among our best MAbs using combinatorial analysis, corresponding to 4 different regions recognized mainly on the NDM-1 protein. This would allow us to develop a new test rapidly by the addition of new antibodies targeting nonmutated regions in the existing LFIA.

Interestingly, another immunochromatographic assay has been developed for the detection of IMP-type metallo-carbapenemases that are highly prevalent in Southeast Asia. This test also presented 100% sensitivity and 100% specificity for the detection of IMP-type carbapenemases in Pseudomonas spp. (28). Moreover, Glupczynski et al. developed a single immunochromatographic test for the detection of both OXA-48-like and KPC carbapenemases (22). Thus, in the near future, we aim to combine different antibodies targeting the five most relevant carbapenemases (OXA-48, NDM, KPC, VIM, and IMP) into a single immunochromatographic assay in order to provide a very powerful and broad-spectrum tool for the detection of CPE.

### Conclusion.

The NDM LFIA was efficient, rapid, and easy to implement in the routine workflow of a clinical microbiology laboratory for the confirmation of NDM-like carbapenemase-producing Enterobacteriaceae. It could complete the existing panel of tests available for the confirmation of NDM carbapenemases, especially in countries with low resources and/or a high NDM prevalence.

## MATERIALS AND METHODS

### Ethics statement.

All experiments were performed in compliance with French and European regulations on the care of laboratory animals (European Community [EC] Directive 86/609, French Law 2001-486, 6 June 2001) and with agreement no. 91-416 delivered to S.S. by the French Veterinary Services and CEA agreement D-91-272-106 from the Veterinary Inspection Department of Essonne (France).

### Reagents.

Biozzi mice were bred at the animal care unit of the CEA (Gif sur Yvette, France). Bovine serum albumin (BSA), Tween 20, isopropyl-β-d-thiogalactoside (IPTG), biotin *N*-hydroxysuccinimide ester, streptavidin, a gold chloride solution, *N*-succinimidyl-*S*-acetyl-thioacetate (SATA), imidazole, and kanamycin (from Streptomyces kanamyceticus) were obtained from Sigma-Aldrich (Saint Quentin Fallavier, France). NdeI and XhoI were obtained from New England BioLabs (Evry, France). Goat anti-mouse (GAM) IgG and IgM polyclonal antibodies were obtained from Jackson ImmunoResearch (West Grove, PA, USA). Protein A-Sepharose (ProsepA) was obtained from Millipore (Guyancourt, France). A serine protease inhibitor [4-(2-aminoethyl)benzenesulfonyl fluoride hydrochloride (AEBSF)] was obtained from Interchim (Montluçon, France). Metal agarose affinity resin (Chelating Sepharose FastFlow) was obtained from GE Healthcare (Vélizy-Villacoublay, France). Enzyme immunoassays (EIAs) were performed with MaxiSorp 96-well microtiter plates (Nunc, Paris, France), and all reagents were diluted in EIA buffer (0.1 M phosphate buffer [pH 7.4] containing 0.15 M NaCl, 0.1% BSA, and 0.01% sodium azide). Plates coated with proteins were saturated in EIA buffer (18 h at 4°C) and washed with washing buffer (0.1 M potassium phosphate [pH 7.4] containing 0.05% Tween 20). Nitrocellulose strips with polystyrene backing were obtained from GE Healthcare (Prima 40). For culture media, Luria broth (LB) and LB agar were obtained from Sigma-Aldrich; tryptone soy agar (TSA) was obtained from Oxoid (Dardilly, France); Mueller-Hinton (MH) agar and URISelect 4 (Uri-4) were obtained from Bio-Rad (Marnes la Coquette, France); and Columbia agar plus 5% horse blood (COH), ChromID ESBL agar, ChromID Carba Smart, and Drigalski (DRIG) agar were obtained from bioMérieux SA (Marcy l'Etoile, France). The Vibra cell 75185 sonicator apparatus was obtained from Bioblock Scientific (Illkirch, France).

### Strains tested.

For the LFIA validation, 175 enterobacterial isolates with characterized β-lactamase content were used to evaluate the NDM LFIA (18). This collection included 55 non-carbapenemase producers and 120 carbapenemase producers, including 27 NDM producers. For the prospective evaluation, 74 enterobacterial clinical isolates with decreased susceptibility to at least one carbapenem were used. These isolates were sent by clinical microbiology laboratories throughout France to the Associated French NRC for CRE.

### Cloning and expression of the NDM-1(29–270) protein in E. coli.

The gene (from E. coli) encoding the protein without a signal sequence from amino acids G29 to R270 (predicted by the SignalIP 4.1 server) was amplified by PCR using forward primer NDM-1 29-270 NdeI (5′-aaaaaCATATGggtgaaatccgcccgacg-3′; lowercase type on the 3′ side of the restriction site [uppercase] represents the gene coding sequence, and “aaaaa” represents irrelevant nucleobases) and reverse primer NDM-1 XhoI-Δstop (5′-aaaaaCTCGAGgcgcagcttgtcggccat-3′). After amplification, the sequence was further cloned into the pET41b vector (Invitrogen, Life Technologies, Cergy-Pontoise, France), using NdeI and XhoI restriction enzymes, allowing the insertion of a polyhistidine tag sequence at the 3′ end of the gene. The inserted gene was verified by sequencing.

E. coli BL21(DE3)/pLysS was then transformed with the recombinant gene. One positive clone was grown in 1 liter of LB with 50 μg/ml kanamycin at 30°C until the optical density at 600 nm (OD_600_) reached 0.6. IPTG (100 μM) was then added to the culture, which was incubated for 4 h with shaking at 30°C. The culture was pelleted by centrifugation at 3,000 × *g* for 15 min at 4°C. The pellet was suspended in 40 ml of solubilizing buffer (50 mM Tris-HCl buffer [pH 8], 100 mM NaCl, 1 mM AEBSF) and frozen at −20°C. After melting, the bacterial suspension was sonicated (3 pulses of 15 s at 14 W) and centrifuged for 15 min at 10,000 × *g*. Imidazole (20 mM final concentration) was added to the supernatant, which was incubated for 2 h with 4 ml of Ni-nitrilotriacetic acid (NTA) agarose affinity resin with shaking at room temperature. The gel was washed with 100 ml of binding buffer (50 mM Tris-HCl buffer [pH 8], 100 mM NaCl, 20 mM imidazole). Elution of the His-tagged protein was performed by incubating the resin for 10 min with 4 ml of solubilizing buffer with 500 mM (final concentration) imidazole, and the operation was repeated 5 times.

The eluted fractions were pooled and dialyzed twice in 2 liters of 50 mM potassium phosphate buffer (pH 7.4). The protein concentration was measured by the absorbance at 280 nm, and purity was assessed by SDS-PAGE (Phast system; GE Healthcare). The recombinant NDM-1 protein was then used to immunize mice, as a standard for the selection of MAb pairs, and to determine the limit of detection.

### Antibody selection for the lateral flow format. (i) Preparation of colloidal gold-labeled NDM-1 antibodies.

Colloidal gold was prepared as previously described (26), 1 ml was centrifuged for 15 min at 15,000 × *g*, and the pellet was suspended in water. One hundred microliters of a 100-μg/ml solution of each MAb (previously produced and selected [see Text S1 in the supplemental material]) in 5 mM phosphate buffer (pH 7.4) was added, and the mixture was incubated for 1 h at 20°C, allowing the adsorption of the MAbs to the surface of the gold particles. One hundred microliters of a solution containing 20 mM phosphate buffer (pH 7.4) and 1% BSA was then added, and the mixture was centrifuged for 15 min at 15,000 × *g*. The supernatant was discarded, and the pellet was suspended in 1 ml of 2 mM phosphate buffer (pH 7.4) and 0.1% BSA, sonicated for a few seconds, and centrifuged for 15 min at 15,000 × *g*. The supernatant was discarded, and the pellet was suspended in 250 μl of a solution containing 2 mM phosphate buffer (pH 7.4) and 0.1% BSA and stored at 4°C in the dark.

### (ii) Strip screening test.

To select the best MAb pairs for the development of the two-site lateral flow immunoassay, a combinatorial analysis was carried out by using each MAb as either a capture or a gold-labeled antibody. Briefly, the strips were prepared by spotting 1 μl of MAb (100 μg/ml in 50 mM phosphate buffer [pH 7.4]) and then dried. One hundred microliters of an NDM-1 solution (50 ng/ml in EIA buffer–0.5% Tween 20) or buffer alone and 10 μl of colloidal gold-labeled MAb were mixed in microtiter plate wells (Greiner) and allowed to react for 5 min before the strip was dipped into the solution. After a 30-min migration, signals were analyzed by the naked eye. The parameters used to select the best MAb pairs were the intensity of the visual signals obtained with an NDM-1 concentration (50 ng/ml) and the absence of a signal without NDM-1 (nonspecific signal).

### (iii) Selection of best pairs.

For this study, we used a conventional strip format. The strips (0.5 cm wide and 4.5 cm long) were composed of 3 parts, (i) a sample pad (standard 14; Whatman) (0.5 cm long), (ii) a nitrocellulose membrane (Prima 40) (2.5 cm long), and (iii) an absorption pad (cellulose-grade 470; Whatman) (1.5 cm long), all attached to a backing card. The detection zone contained immobilized goat anti-mouse antibodies as a control line and an anti-NDM-1 MAb as a test line (1 mg/ml in 50 mM sodium phosphate buffer [pH 7.4]) dispensed at 1 μl/cm by using an automatic dispenser (Biojet XYZ 3050; BioDot, England). After drying for 1 h at 37°C in an air oven, the membrane was incubated with a blocking solution (phosphate-buffered saline [PBS] [pH 7.4] containing 0.5% BSA) for 30 min at room temperature (RT). The membrane was washed twice with deionized water, incubated for 20 min at RT in a preserving solution (PBS containing 0.1% Tween 20 and 7.5% glucose), and then dried for 20 min at 37°C in an air oven. After the absorption pad and the sample pad were fixed to the top and the bottom of the membrane, respectively, the card was cut into 5-mm-wide strips by using an automatic programmable cutter (CM4000 Guillotine cutting system; BioDot).

Each selected pair of antibodies was evaluated in the LFIA format using serial dilutions (1 × 10^8^, 2 × 10^7^, 4 × 10^6^, 8 × 10^5^, and 1.6 × 10^5^ CFU/ml) of NDM-producing bacteria in extraction buffer {100 mM Tris-HCl (pH 8), 0.15 M NaCl, 0.1% BSA, 0.5% Tween 20, 1% 3-[(3-cholamidopropyl)-dimethylammonio]-1-propanesulfonate (CHAPS)}. One hundred microliters of this solution was mixed for 5 min with 10 μl of the conjugate antibody before the strip was dipped (for each pair of antibodies).

### NDM LFIA evaluation and use.

The NDM LFIAs (strip plus cassette) were manufactured by NG Biotech (Guipry, France) using our MAbs. The 175 strains to be tested were grown on MH agar for 16 h at 37°C. Ten strains were also grown under the same conditions on TSA, Uri-4, COH, ChromID ESBL, ChromID Carba Smart, and DRIG agar plates. Using a 1-μl inoculation loop, a single colony was resuspended in 150 μl of extraction buffer (lysis step) and vortexed for a few seconds, and subsequently, 100 μl was dispensed onto the cassette. The results were read by eye after 15 min of migration by monitoring the appearance of a red band specific for NDM-1, along with a band corresponding to the internal control. This protocol is similar to the one recommended for the future commercial NDM LFIA.

### Limit of detection with recombinant NDM-1 and NDM-1-producing enterobacterial isolates.

Serial dilutions (50,000, 16,666, 5,555, 1,852, 617, 206, 68, and 0 pg/ml) of the recombinant NDM-1 protein or of one NDM-1-producing K. pneumoniae strain (6 × 10^8^, 1.2 × 10^8^, 2.4 × 10^7^, 4.8 × 10^6^, 9.6 × 10^5^, and 1.9 × 10^5^ CFU/ml) were performed with extraction buffer or LB medium, respectively. Bacterial dilution vials were centrifuged for 20 min at 5,000 × *g*, the supernatant was carefully discarded, and the pellet was suspended in extraction buffer (same volume as LB). Consecutively, 100 μl of each solution was dispensed onto the cassette and allowed to migrate. Results were read by eye after 15 min.

## Supplementary Material

Supplemental material
