# Supplementary material for: Development and Validation of a Lateral Flow Immunoassay for Rapid Detection of NDM-Producing Enterobacteriaceae
Source: J Clin Microbiol. 2017 Jun 23;55(7):2018–29. doi: 10.1128/JCM.00248-17 (PMC5483903; doi:10.1128/JCM.00248-17)
Supplement: Supplemental material [file supp_55_7_2018__index.html]

Supplemental material 

# Development and Validation of a Lateral Flow Immunoassay for Rapid Detection of NDM-Producing Enterobacteriaceae

## Supplemental material

- Supplemental file 1 -

  Text S1 (Production and characterization of anti-NDM-1 MAbs)

  PDF, 75K
