# Supplementary material for: Development and Validation of a Lateral Flow Immunoassay for Rapid Detection of NDM-Producing Enterobacteriaceae
Source: J Clin Microbiol. 2017 Jun 23;55(7):2018–29. doi: 10.1128/JCM.00248-17 (PMC5483903; doi:10.1128/JCM.00248-17)
Supplement: Supplemental material [file JCM.00248-17_zjm999095538s1.pdf]

Production and characterization of anti-NDM-1 mAbs.

Ten-week-old Biozzi mice were immunized by intraperitoneal injection of purified recombinant NDM-1 (50 µg) as previously described (1). The immune response was followed by measurement of the anti-NDM-1 antibodies in sera using biotin-labeled NDM-1 as antigen. The two mice with the highest antibody titers were selected for mAb production. Spleen cells were fused with NS1 mouse myeloma cells (2), and specific anti-NDM-1 antibodies in myeloma culture supernatants were detected using the same immunoenzymatic test as the one previously used for the polyclonal response evaluation (3). After cloning of the cells according to Kohler and Milstein (4), mAbs were produced in and purified from culture supernatant by affinity chromatography using protein A and dialyzed in 0.05 M phosphate buffer pH 7.4.

1. Simon S, Demeure C, Lamourette P, Filali S, Plaisance M, Créminon C, Volland H, Carniel E. 2013. Fast and Simple Detection of Yersinia pestis Applicable to Field Investigation of Plague Foci. PLoS ONE 8.

2. Grassi J, Frobert Y, Lamourette P, Lagoutte B. 1988. Screening of monoclonal antibodies using antigens labeled with acetylcholinesterase: Application to the peripheral proteins of photosystem 1. Anal Biochem 168:436–450.

3. Frobert Y, Grassi J. 1998. Screening of Monoclonal Antibodies Using Antigens Labeled with Acetylcholinesterase, p. 57–68. *In* Immunochemical Protocols. Humana Press, New Jersey.

4. Köhler G, Milstein C. 1975. Continuous cultures of fused cells secreting antibody of predefined specificity. Nature 256:495–497.
